# Targeting Endothelial Barrier Dysfunction Caused by Circulating Bacterial and Mitochondrial N-Formyl Peptides With Deformylase

**DOI:** 10.3389/fimmu.2019.01270

**Published:** 2019-06-06

**Authors:** Patricia Martinez-Quinones, Amel Komic, Cameron G. McCarthy, R. Clinton Webb, Camilla Ferreira Wenceslau

**Affiliations:** ^1^Department of Surgery, Medical College of Georgia, Augusta University, Augusta, GA, United States; ^2^Department of Physiology, Medical College of Georgia, Augusta University, Augusta, GA, United States; ^3^Department of Physiology and Pharmacology, University of Toledo, Toledo, OH, United States

**Keywords:** sepsis, SIRS, trauma, DAMPs, formyl peptide receptor-1, deformylase, endothelium

## Abstract

Despite recent advances in our understanding of the mechanisms underlying systemic inflammatory response syndrome (SIRS) and sepsis, the current therapeutic approach to these critically ill patients is centered around supportive care including fluid resuscitation, vasopressors and source control. The incidence of SIRS and sepsis continues to increase in the United States and patients die due to failure to respond to the traditional therapies of nitric oxide blockade, adrenergic agonists, etc. Bacterial and mitochondrial N-formyl peptides (NFPs) act as damage-associated molecular patterns and activate the innate immune system through formyl peptide receptors (FPR) located in immune and non-immune cells, including the vascular endothelium. The resulting inflammatory response manifests as capillary leak, tissue hypoperfusion and vasoplegia, partially due to endothelium barrier breakdown. Potential strategies to prevent this response include decreasing NFP release, breakdown of NFPs, and blocking NFPs from binding FPR. We propose the use of deformylase, the degrading enzyme for NFPs, as potential therapeutic approach to prevent the deleterious effects of NFPs in SIRS and sepsis.

## Introduction

Systemic inflammatory response syndrome (SIRS) and sepsis are major causes of morbidity and mortality in the United States. Sepsis and its related morbidity and mortality is considered the most expensive condition treated in the United States by the Agency for Healthcare and Research Quality, costing an approximate 20 billion dollars yearly (1–3). Despite advances in critical care, few targeted therapies have been developed for patients with SIRS, sepsis and multi-system organ dysfunction (MSOD) (4). While sepsis has been recently defined as life-threatening organ dysfunction caused by a dysregulated host response to infection (5), SIRS may reflect an appropriate host response, which can be caused by various pathologic insults, including trauma and tissue injury (5, 6). Trauma and tissue injury lead to a sepsis-like clinical picture, “microorganism-free sepsis,” which clinically mimics sepsis, although no microbial pathogen is present.

SIRS and sepsis are complex inflammatory syndromes that involve activation and amplification of the innate immune system through receptor interaction with “danger signals” or “target molecular motifs.” In the case of traumatic tissue injury, this response can be attributed to endogenous molecules known as damage-associated molecular patterns (DAMPs) (7). DAMPs are released when tissue injury occurs and trigger an innate immune response similar to that of pathogen-associated molecular patterns (PAMPs) (8–10). PAMPs are conserved pathogenic structures such as bacterial DNA, bacterial cell wall components and bacterial N-Formyl peptides (fMLP). DAMPs are evolutionarily conserved endogenous molecules not normally found in the circulation such as mitochondrial DNA, mitochondrial N-Formyl peptides (FMITs), basement membrane fragments, histones and heat shock proteins. Innate immune system activation through pattern recognition receptors (PRRs), such as formyl peptide receptors (FPRs) and toll-like receptors (TLRs) (11), leads to the production of high levels of proinflammatory cytokines (tumor necrosis factor (TNF-α), interleukin (IL)-6, interferon gamma (IFN-γ), etc.) causing systemic derangement and cardiovascular collapse (12–14). The sharing of cellular pathways by which DAMPs and PAMPs act may cause the downstream immune responses to SIRS and sepsis to be indistinguishable from one another. This may thus explain the similarity in clinical responses to infective and non-infective challenges.

Increased circulating levels of mitochondrial DAMPs have been associated with increased morbidity and mortality in critically ill adult patients (7, 15, 16). Our laboratory has recently demonstrated that trauma patients with SIRS have increased levels of circulating mitochondrial NFPs compared to control trauma patients without clinically diagnosed SIRS (17). Furthermore, trauma patients who went on to develop sepsis during their hospitalization where found to have even higher levels of mitochondrial NFPs in their plasma, compared to those of SIRS patients (17). In a rat model of hemorrhagic shock, mitochondrial NFPs (N-formyl-Met-Met-Tyr-Ala-Leu-Phe) induced severe hypotension, hyperthermia, lung injury, microvascular thrombosis and vascular leakage (18).

Elevated DAMPs lead to inflammation and end-organ damage *in vitro* and *in vivo* (7, 14, 17, 19). In a murine model of acute lung injury with tracheal infusion of mitochondrial NFPs, we showed a concentration-dependent contraction of the trachea, bronchi and bronchioles, which was decreased with FPR-1 antagonist administration (17). Nonetheless, the underlying mechanisms by which NFPs affect non-immune cells and lead to SIRS after traumatic injury are still being investigated. Similarly, targeted degradation of mitochondrial DAMPs *in vitro* has offered a potential therapeutic alternative for the treatment of these devastating diseases, especially in patients that do not respond to traditional therapies (20).

## Vascular Leakage as a Link Between SIRS and Sepsis

SIRS and sepsis are different manifestations of an underlying complex pathophysiology with many etiologies. Both SIRS and sepsis can lead to multi-system organ dysfunction and potentially death (21). One of the major characteristics of these conditions is the breakdown of vascular endothelial barrier function (4, 6, 22), which can result in hemodynamic collapse and shock. An increase in vascular permeability (or vascular leakage) leads to progressive subcutaneous and body-cavity edema, clinically referred to as anasarca (4). Whether endothelial barrier dysfunction is a cause or an effect of the disease process underlying SIRS and sepsis has yet to be determined. Nonetheless, understanding the molecular mechanisms causing endothelial barrier breakdown might lead to new pharmacologic approaches for its prevention and ultimately to an innovative treatment.

An increase in vascular endothelium permeability, secondary to endothelial barrier dysfunction, has been previously associated with pro-inflammatory factors such as reactive oxygen species, TNF-α, IL-1, IL-2, and IL-6 (23), known to be elevated in SIRS and sepsis. However, pharmacological interventions that inhibit these molecules have not been successful at preventing or reversing endothelial damage (22). Further, inhibition of TLR-4 with the antagonists E5564 and TAK-242 showed no effects on 28-days mortality reduction in sepsis (24, 25). Similarly, polyclonal intravenous immune globulin administration has shown variable results; however, randomized trials showed no benefits when compared to placebo (26–28). Additionally, use of a recombinant, non-glycosylated human IL-1 receptor antagonist also showed no improvement in patients with severe sepsis and septic shock (29, 30).

Due to the lack of understanding of the molecular mechanisms underlying endothelial barrier dysfunction, therapies targeting vascular leakage in SIRS and sepsis are not currently available. Our aim is to better understand the underlying mechanisms of how bacterial and mitochondrial NFPs lead to vascular leakage, and to devise strategies which may specifically target NFP pathways. With this knowledge we can devise potential strategies which may target NFPs, breakdown of circulating NFPs and/or preventing NFPs from binding its target receptor, FPR-1.

The pro-inflammatory nature of NFPs and their critical role in initiating pathogenic and sterile inflammatory responses makes them an appealing therapeutic target. While activation of the innate immune system is necessary for clearance of the offending bacterial organism or injured tissue, it is unknown how much NFP is needed to potentiate the inflammatory response and alter this response from adaptive to maladaptive. Bacterial NFPs all contain a conserved secondary structure, allowing for a large pool of pathogens to activate FPR-1 with similar affinity and elicit a similar response (31). FPR-1 activation by fMLP (a bacterial NFP) triggers neutrophil chemotaxis, diapedesis, and degranulation (32–34) and neutrophils deficient in FPR-1 display impaired chemotaxis (35). As mentioned above, we have previously shown that fMLP induce vascular leakage and exacerbate vasodilatation in rat mesenteric resistance arteries, and that Cyclosporin-H (CsH), an FPR-1 antagonist, inhibited this response (14).

## FPR-1 SIGNALING and Innate Immune System Activation

FPR-1 has differential expression in various immune cells (e.g., dendritic cells, neutrophils, mast cells) and non-immune cells (e.g., somatic cells of the cardiovascular system, including the endothelium) (33). FPR-1 detects evolutionarily conserved molecules found in bacteria and recognizes the bacterial origin of mitochondria (7, 14, 36). FMIT exposure to vessels also induces FPR-1-mediated vascular relaxation that is inhibited by CsH (14).

FPR belongs to G-protein coupled receptor (GPCR) family and important components of the innate immune system (4). FPRs were first discovered in neutrophils and are now known to be comprised of 3 members in humans (FPR-1, FPR-2, and FPR-3) and 8 in mice (37, 38). Each member of the FPR family has differential expression and binding affinities, with FPR-2 and FPR-3 having lower affinity and a greater number of ligands (39, 40). FPR-1 activation by NFPs triggers immune reactions in neutrophils, monocytes and macrophages (41). FPR-1 is a high affinity binding site for the NFP sequence fMLP, with the ability to recognize even small picomolar concentrations (42). FPR-1 activation in phagocytic cells triggers degranulation, pro-inflammatory cytokine and chemokine production and reactive oxygen species generation (41). Contrary to FPR-1, FPR-2 can bind a variety of ligands, although with lower affinity, including select bacterial NFPs and non-formylated ligands like annexin-1, resolvin D1 and lipoxin A4, among others (33, 42, 43). FPR-2 has been observed to prevent excessive inflammatory responses in animal models of meningitis and Alzheimer's disease (44, 45). FPR-3 function remains unclear, as it is not present in hematopoietic cells and its ligands do not overlap with FPR-1 and FPR-2 (46, 47). To our knowledge there are no data to suggest FPR-3 involvement in endothelium integrity.

FPR-1 is also expressed in non-immune cells, suggesting that FPR-1 serves other functions besides sensing targeted molecular motifs (48). FPR-1 is essential for vascular homeostasis, as shown in our recent work where FPR-1 was found to be fundamental for myogenic vascular contraction under physiological conditions (49). FPR-1 has been implicated in cell growth and proliferation in tumorigenesis (50, 51). FPR-1 activation in immune and non-immune cells triggers intracellular signal transduction pathways responsible for transcriptional regulation, cytoskeletal reorganization, superoxide production, and exocytosis of granules (34, 52, 53). Activation of FPR-1 signaling contributes to the physiological defense against danger signals and makes FPR-1 an attractive therapeutic target.

The identification of selective FPR antagonists has allowed for the continued discovery of this receptor family interactions and potential implications in disease. Interestingly, some pathogens produce FPR antagonists. For instance, the pertussis toxin from *Bordetella pertussis* is a potent inhibitor of GPCR-mediated leukocyte chemotaxis by inactivating the Gα_i_ protein of FPR (42). The most widely used FPR-1 antagonist Cyclosporin H (CsH) is a high affinity inverse agonist, selective for FPR-1, which “locks” FPR-1 into an inactive conformation (54, 55). Due to the intrinsic relationship between FPR-1, the actin cytoskeleton and transcription regulation, antagonizing this receptor is a problematic approach (37). In a model of pneumococcal meningitis, FPR-1 deficient mice were found to have increased bacterial burden, increased neutrophil infiltration and elevated mortality rates (44). Oldekamp and colleagues also showed that FPR-1 deficient microglial cells have attenuated cell viability after bacterial exposure to *S. pneumoniae* and *N. meningitidis* (44). FPR-1 deficient mice also have increased susceptibility to *Listeria monocytogenes* as evidenced by increased bacterial load in the spleen and liver (56). Increased knowledge of the direct importance of FPR-1 in physiological and pathophysiological conditions is still needed. Challenges exist in targeting FPR-1 directly because of its intrinsic functional properties and its ability to mediate both pro-inflammatory and anti-inflammatory effects depending on the activating ligand (57). Furthermore, since its absence leads to an enhanced inflammatory response, other approaches to targeting FPR-1 signaling must be considered for potential therapeutic applications.

When FPR-1 is activated, it mediates chemotaxis (58), signals intracellular cascades (59), induces cell cytoskeleton rearrangement (48), and may act as a mechanosensor (49, 60). In phagocytic cells, FPR-1 blockade in these cells impairs their function, prevents their migration to sites of infection and decreases bacterial clearance (35). FPR-1 activation in non-immune cells may occur through neutrophil-dependent and/or neutrophil-independent pathways (19). In accordance with prior studies, we showed that FPR-1 is not only present in immune cells but also on vascular endothelial and vascular smooth muscle cells (18, 48, 61). FPR-1 expression in somatic vascular cells is consistent with the theory that each tissue and cell type can tailor its own immune response. FPR-1 mediated generation of pro-inflammatory cytokines, chemokines, and adhesion molecules has been extensively studied in immune cells, specifically neutrophils. However, whether or not the same signaling pathways and downstream effects of FPR-1 activation occur in vascular endothelial cells has yet to be determined.

In the pathophysiologic states of SIRS and sepsis, disturbances in the microcirculation are associated with worse clinical outcomes, and this occurs independent of macrohemodynamic changes. Factors proposed to contribute to this capillary leakage, and disturbances in blood flow, include platelet aggregation, endothelial cell injury, and increased microvascular permeability with accompanying interstitial edema, among others (62). Microvascular injury compromises capillary blood flow, leading to capillary flow cessation and potentially causing further hypoxic tissue injury. Endothelial cell activation, independent of leukocyte activation, triggers a localized inflammatory response and exacerbates microvascular leakage (62).

Vascular endothelium is one of the tissues most affected by sepsis and traumatic injury, either by trauma itself and/or the inflammatory reaction after trauma. An intact vascular endothelium is necessary to maintain barrier function, osmotic balance, solute transport, and to prevent pathogens and molecules from reaching the sub-endothelial connective tissue (22). The endothelial cell lining of the vasculature constitutes a semi-permeable barrier between the intravascular and the interstitial space. Under physiological conditions, activation of FPR-1 in vascular endothelial cells is necessary to allow neutrophil and monocyte migration to sites of inflammation, allowing for reduction of bacterial burden (35). However, an exacerbated inflammatory response may lead to endothelial cell apoptosis and necrosis, detachment and loss of endothelium barrier function (63). Increase in endothelium permeability allows for immune cell infiltration, interstitial edema, and potentiation of end-organ damage. Breakdown of the endothelium lining may hinder bacterial clearance as an intact endothelial cytoskeleton is necessary for paracellular transport and leukocyte transmigration (64). Disruption of this barrier integrity manifests as hyper-permeability which is associated with many systemic diseases, including SIRS and sepsis.

The integrity of vascular endothelium is influenced by an intact endothelial cytoskeleton. FPR-1 activation leads to cytoskeletal rearrangement resulting in endothelial cell contraction via actin-myosin interaction and actin polymerization (48). However, the intracellular molecular mechanisms by which NFPs, from bacteria and mitochondria, lead to vascular injury and endothelial barrier breakdown remain incompletely understood. Our working hypothesis is that NFPs, whether exogenous or endogenous, lead to increased vascular endothelial cell permeability through FPR-1 activation, causing downstream actin cytoskeletal rearrangement and endothelial contraction.

## Deformylase: a New Pharmacological Tool to Prevent Endothelium Barrier Dysfunction in SIRS and Sepsis?

There are still major gaps in our understanding of the underlying pathophysiology of trauma-induced SIRS and sepsis. Particularly how DAMPs and PAMPs interaction with PRRs give rise to the multiple cytokines and chemokines produced during SIRS and sepsis, and the subsequent physiological consequences. The inability to treat or prevent trauma-induced SIRS and microbial sepsis may be due to our limited understanding of the underlying molecular mechanisms causing endothelial dysfunction and vascular leakage (48). Given that loss of FPR-1 function could affect appropriate innate immune system response, it should be important to identify means for restoring or bypassing deficiencies in FPR-1 signaling.

FPR-1 antagonists have the potential to inhibit the functional intrinsic properties of this receptor in endothelial cells. However, since FPR-1 ligands involved in eliciting innate immune response can be discriminated, this may offer an opportunity to prevent deleterious downstream FPR-1 signaling. Targeting DAMPs and PAMPs and their receptors is a promising therapeutic strategy for the management of inflammatory pathologies (65). For instance, cell-free mitochondrial DNA is currently being studied as a therapeutic target in myocardial infarction (66) with the use of Endonuclease III, an enzyme that digests DNA. Additionally DNase, another family of enzymes that digests DNA (67), has shown the potential to degrade mitochondrial DNA in *in vitro* studies (68). DNase has been shown to be elevated in the systemic circulation following traumatic injury (69) and may have potential targeting against mitochondrial DNA. However, no enzymes with the potential to digest NFPs have been identified to be present in the systemic circulation.

Peptide deformylase, a metalloenzyme, has the inherent activity to degrade NFPs before they bind FPR-1, and may in fact serve as a potential pharmacologic agent for the treatment of trauma-induced SIRS and sepsis. Deformylase removes the formyl group at the N terminus of nascent polypeptides in bacteria and mitochondria (70). This enzyme, acting as a monomer, binds a metal ion and catalyzes the reaction: N-formyl-L-methionine + H_2_O = formate + methionyl peptide (71). Deformylase is essential in prokaryotes and it was previously thought that deformylase was unique to bacteria, hence it has been a target for the creation of antibacterial agents against its activity (70, 72). Deformylase was previously targeted by antibacterial agents after the finding that its inhibition in *E. coli* was bactericidal (73–75). These agents were found to target both bacterial and human mitochondrial deformylase, as it was later discovered that the three-dimensional structure of deformylase is evolutionarily conserved (76). This led to the identification of deformylase homologs in eukaryotes, including human mitochondria (72, 77). To date, its function in human mitochondria is not well-defined. What is known, is that human mitochondrial deformylase is necessary for translation initiation of respiratory complexes; therefore its inhibition disrupts mitochondrial function (72). Some peptide deformylase inhibitors with activity against bacterial peptide deformylase have been isolated and studied in Phase I clinical trials with variable but non-clinically significant adverse effects (70, 72, 78). The deformylase inhibitors BB83698 and LBM415 studied in phase I clinical trials in humans were ultimately found to have poor selectivity (78). The first deformylase inhibitor to be studied in a clinical trial was LBM415 (Novartis Pharmaceuticals), its oral administration led to the unexpected finding of methemoglobinemia in study participants; these results were later confirmed with *in vitro* and *in vivo* animal studies (79). Deformylase inhibitors impair not only bacterial deformylase but also human mitochondrial deformylase and prevent mitochondrial translation and oxidative phosphorylation (80, 81).

**Figure 1:**
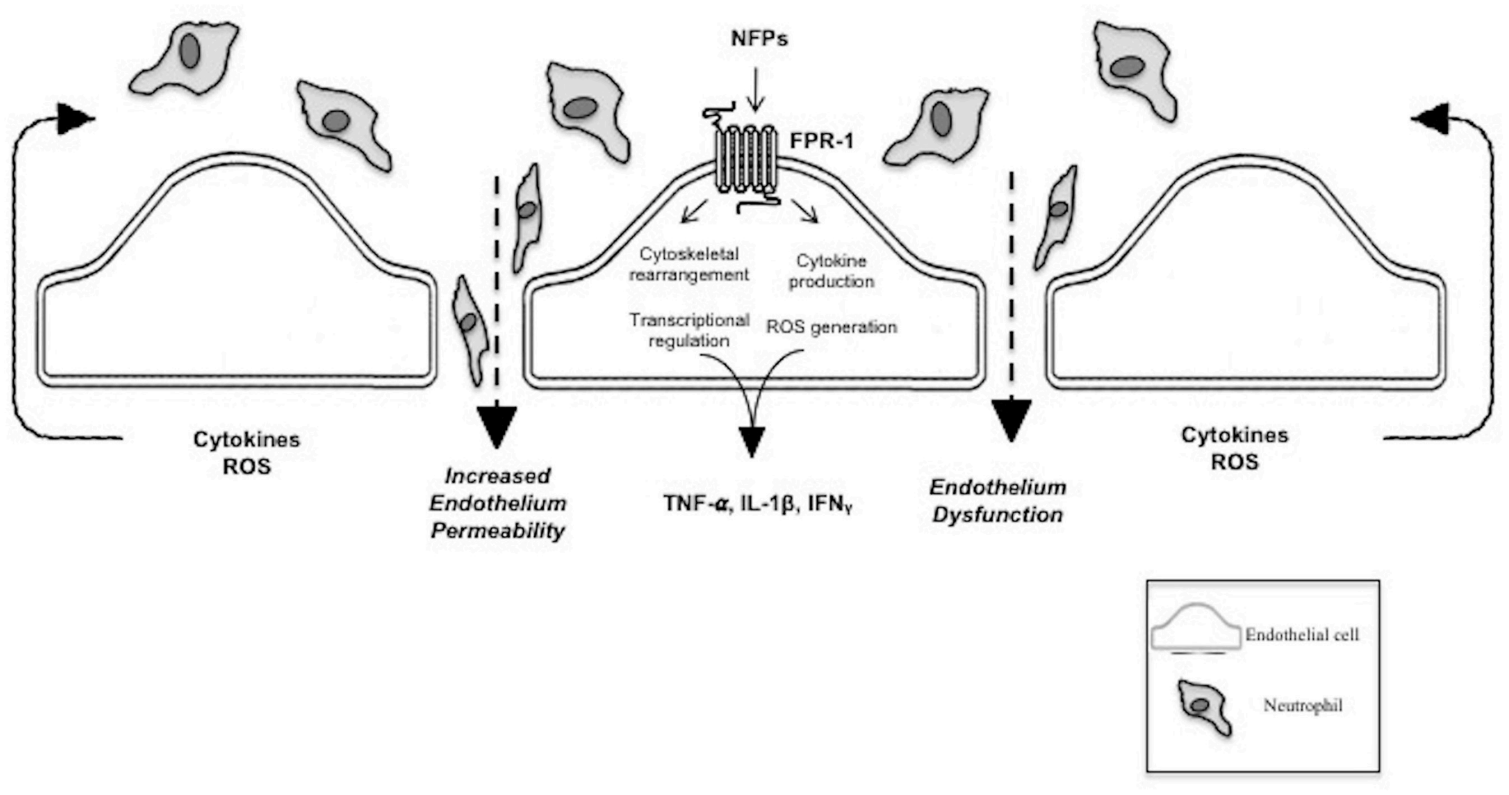
N-Formyl peptide mediated pathophysiology of SIRS and sepsis. This figure shows the pathogenic effect of NFPs on the vascular endothelium via FPR-1 activation. FPR-1 activation results in dysfunction of the vascular barrier allowing infiltration of immune cells and molecules into the interstitial and extravascular space. NFPs, bacterial and mitochondrial N-Formyl peptides; FPR-1, formyl peptide receptor-1; TNF-α, tumor necrosis factor alpha; ILF-1β, interleukin-1 beta; IFNγ, Interferon gamma; ROS, reactive oxygen species.

The proteolytic effects of deformylase make it an attractive target for drug development. To date peptide deformylase itself has not been studied as a therapeutic option for the management of SIRS or sepsis. Our group has investigated the links between trauma, vascular collapse and sepsis (Figure 1), and our results suggest that NFPs and FPR-1 may serve as that link (14, 17, 18, 48). We have recently found that deformylase is a potent treatment for sepsis in a murine cecal ligation and puncture model of intraperitoneal sepsis and an *in vitro* cell culture model of SIRS (unpublished) (US Provisional Patent Application 62/790, 185 “Methods and Compositions of Treating Sepsis and Systemic Inflammatory Response Syndrome”).

**Figure 2:**
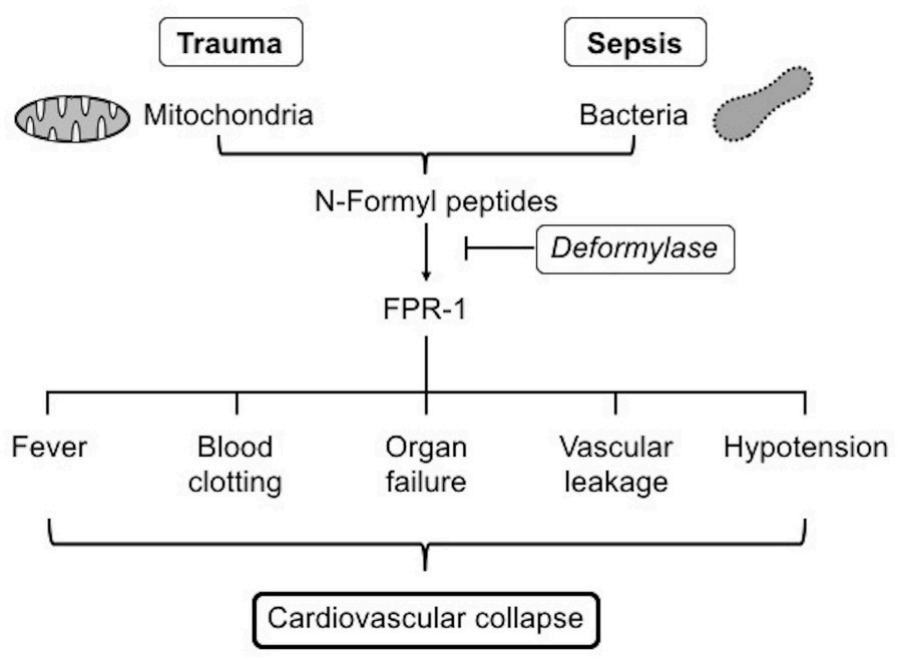
FPR-1, formyl peptide receptor-1.

Based on previous findings, it is reasonable to speculate that administration of deformylase, as the degrading enzyme for both bacterial and mitochondrial NFPs, may serve a therapeutic role in preventing FPR-1 activation and its subsequent endothelium barrier dysfunction and vascular leakage (Figure 2). To assess the potential therapeutic strategy of NFP degradation in SIRS and sepsis, we still need to understand the settings in which activation or inhibition of FPR-1 is beneficial or detrimental to injury repair and pathogenic clearance. Furthermore, the quantity of NFPs needed to potentiate an inflammatory response and tilt the balance from an adaptive response to a maladaptive response is unknown.

## Implications

Despite advances in the care of critically ill patients, further improvements in the field are limited by a lack of knowledge of the underlying pathophysiology in SIRS and sepsis. Our current therapeutic approach is centered around supportive care including fluid resuscitation, vasopressors and source control with antibiotics or surgical intervention. The endothelium plays a central role in initiating, promoting and exacerbating the overwhelming inflammatory response. The molecular mechanisms involved in endothelium barrier dysfunction, vascular leakage, and cardiovascular collapse are still being discovered. Bacterial and mitochondrial NFPs, and their activation of FPR-1 in immune and non-immune cells, may serve as a link to the underlying pathophysiology of trauma-induced SIRS and sepsis. The potent pro-inflammatory nature of NFPs and their role in initiating sterile and infective inflammation make them an attractive therapeutic target. The development of therapeutic agents to neutralize the inflammatory effects of NFPs promises to dramatically improve trauma management. The potential use of deformylase itself holds translational value for further pre-clinical and clinical testing considering that very few, if any, targeted therapies are currently available for the treatment of trauma-induced SIRS and sepsis.

Much is still yet to be determined about the consequences of NFPs and FPR-1 receptor activation, especially in vascular somatic cells. Major barriers still exist in the search for immune-based interventions in trauma and sepsis: (1) understanding the underlying molecular mechanisms triggering SIRS, (2) identification of potential biomarkers that could serve as therapeutic targets, (3) identification of meaningful pre-clinical and clinical endpoints other than death, and (4) clinically, establishment of early intervention and logistics for consent waived trials. Our current work and hypotheses may have implications in the management of SIRS and sepsis patients, especially in those who are non-responders to traditional vasopressors. FPR-1-NFP interaction may serve as the missing link between host-derived danger signals, inflammation and vascular dysfunction in SIRS and sepsis.

## Author Contributions

PM-Q, AK, and CGM co-wrote the manuscript. RCW critically reviewed the manuscript and contributed to Figures 1, 2. CFW co-wrote the manuscript and supervised the project. All authors agree to be accountable for the content of the work.

### Conflict of Interest Statement

The authors declare that the research was conducted in the absence of any commercial or financial relationships that could be construed as a potential conflict of interest. The handling editor is currently co-organizing a Research Topic with one of the authors CFW, and confirms the absence of any other collaboration.
